# Topics of the Month

**Published:** 1895-05

**Authors:** 


### TOPICS OF THE MONTH.

THE JOURNAL is gratified beyond measure at the generous treatment it is receiving at the hands of its contemporaries and the Buffalo newspapers in reference to its approaching semicentennial anniversary. Not only have they written us personal letters in great numbers, filled with pleasant words of commendation for both the JOURNAL and ourselves personally, all of which we shall acknowledge by letter in due time, but they have overwhelmed us with kindness in their published notices. For all of this we beg them to accept our appreciative thanks.

It may not be out of place for us to say in regard to our proposed jubilee celebration, that it is not our intention to present barely one elaborate anniversary number, profusely illustrated and otherwise expensively published, but we shall issue to our patrons a journal permanently enlarged and improved in every department, so that our subscribers, advertisers, exchanges, general readers and numerous friends will be benefited during all the remaining years which may be allotted to our journalistic existence. We believe this to be a more fitting testimonial to the substantial support we have received from our patrons during all these years, than to present them with a single jubilee number published on the lines hereinbefore indicated. The JOURNAL will be enlarged at the beginning of the next volume, August, 1895.

To one and all whose kind words have given us so much encouragement we again tender our most grateful thanks.

---

THE establishment of free public baths has been occupying the attention of the health commissioner of Buffalo for some time. Many cities have already built institutions of this kind and their citizens are now reaping the sanitary benefits therefrom. While the common council of Buffalo has been delaying in the matter, the legislature has passed a bill, that has already become a law, making it mandatory upon cities of the first and second class to build free baths, while it permits cities below 50,000 inhabitants to raise money for a like purpose. We hope soon to see free public baths in full operation in Buffalo.

---

AN INTERESTING freak of nature has lately occurred in New York. Two children were born to the wife of J. Koehler, of No. 342 East 42d street, in that city on Monday, April 15, 1895, that are attracting considerable attention in professional as well as non-professional circles. They are both girls and are attached to one another at the lower part of the spinal column and upper part of the pelvis. It is believed that the sacrum and coccyx are identical in both bodies. A singular feature of this case is, that the children, though joined at the back, are able to face to the front, owing to the elasticity of the structures at the point of union. They each have complete sexual organs and extremities that are separate and distinct. The father is thirty-five years old and the mother thirty. Both are reported to be healthy. At last accounts the twin babies and their mother are doing well. Not since the wonderful Siamese twins, who grew into advanced life, both married and had children, has such a phenomenon been recorded as this one of the Koehler babies. Considerable interest is manifesting in the probabilities of a surgical procedure being competent to separate these children without jeopardizing life.

---

OUR esteemed contemporary, the *Maryland Medical Journal*, in recently commenting about state boards of medical examiners, makes some statements that we feel compelled to challenge. There is, perhaps, wholesome truth in its assertion that these boards have been made necessary by the lack of moral tone in college faculties. But when it asserts that there is an element of injustice in compelling a man to put himself a second time in peril of his future medical life after he has won his college diploma, we are of the opinion that it is a criticism which will not meet with general approbation.

Does our Maryland contemporary think it an injustice for a physician to be called upon to take a separate examination if he desires to enter the army or navy? Does it also think there is injustice in requiring separate examinations for hospital appointments? Surely the people of the state at large are entitled to quite the same degree of protection as our soldiers or sailors, or patients in hospitals. If it is wrong for the state to require the examination in the one case, it is equally so for the authorities to demand it in the others. We are sorry to take issue with the *Maryland Medical Journal*, which is so seldom at fault in its judgment and opinions.

---

THE dress reform agitation as applied to women has had full swing for some time in the medical journals. Suppose, therefore, though admitting there may be need enough for such reform from a sanitary standpoint, we let the women rest for a while and give the men a chance to be heard on the same question; for assuredly there is some need of improvement in the attire of the sterner sex. With the unsightliness of some of the present fashions we may not contend in this place, except insofar as they relate to health. But there are one or two points that bear strongly on the latter question that we wish to speak of in condemnation. The tight shirt band and high standing collar are not only abominations, but contribute to provoke headaches, disturbances of vision, vertigo and other cerebral perturbations. These reacting on the digestive and circulatory systems, may lay the foundation of, or excite into action, innumerable maladies that otherwise might go unexplained in their etiology.

Another unsightly as well as injurious fashion prevails with reference to foot dress. The "toothpick" shoe is not only a mechanical deformity, but it provokes corns, bunions and other foot diseases that are not to be regarded lightly.

Furthermore, if men would stand erect in submitting to the measurements of their tailors, they would obtain suits that would permit of better chest expansion and thus increase the cubic air space allotted to their breathing organs. These are a few injurious features of the prevailing fashions for men that need correction.

———

The so-called faith-cure has met with a reverse in Dayton, O., that ought to open the eyes of believers in the fad. The *Cincinnati Lancet-Clinic*, of March 30, 1895, devotes an editorial to the episode from which we learn that the case referred to was that of a child suffering from tuberculous meningitis. Through the influence of relatives it was treated by faith-cure methods until death occurred. We cannot do justice to the case in a more befitting manner than to quote the *Lancet-Clinic's* own words in commenting on the affair, which are as follows:

> During the course of the disease those having charge of the little patient did not comprehend or know the nature of the malady, but persisted in the idiotic delusion that there is no such thing as pain and disease. Every day during the early history of the case the child was taken out riding as though nothing was the matter. From first to last no medicines were given, or other treatment that would alleviate the sufferings of the patient. In mercy, death came to the child's relief. The next link in the history was a coroner's inquest and a post-mortem examination, which revealed the fatal nature of the malady.
>
> Here was an instance of cruelty and bigotry which makes some parties guilty of a most heinous crime. The broad charity and humanitarianism of the present day is sufficient to provide every man, woman and child with support and scientific medical attention during sickness. In this instance no attempt was made to secure the advantages of what was to be had for the asking. These people were not financially poor. They were bigots to the extent of criminality. There must be a law somewhere on the statute books of the state that will take cognizance of such procedures. In this instance there should be a rigorous enforcement of its provisions, because, so far as we are able to learn, there were not even shadows of extenuating conditions and circumstances.
>
> Unfortunately, there are some people so constituted as to be prone to the contraction of certain diseases; others are of a neurotic temperament and consequently more or less visionary and hysterical. These visionary, hysterical people are the ready victims of such nonsensical perversions as faith-cure methods and constitute the capital stock in trade of a limited number of spiritualistic fanatics and neurotic perverts. The sun-dance of red-skin savages, with its accompanying sacrifices of human life, is equally justifiable with faith-cure and Christian science practices.

These words deserve to be carefully weighed and pondered by every believer in this rash nonsense which would be ridiculous if it were not so often criminal.

